# The Systematic Review Data Repository (SRDR): descriptive characteristics of publicly available data and opportunities for research

**DOI:** 10.1186/s13643-019-1250-y

**Published:** 2019-12-20

**Authors:** Ian J. Saldanha, Bryant T. Smith, Evangelia Ntzani, Jens Jap, Ethan M. Balk, Joseph Lau

**Affiliations:** 1 Department of Health Services, Policy, and Practice, Center for Evidence Synthesis in Health, Brown University School of Public Health, 121 South Main Street, Box G-S121-8, Providence, RI 02903 USA; 2 Department of Epidemiology, Center for Evidence Synthesis in Health, Brown University School of Public Health, 121 South Main Street, Box G-S121-8, Providence, RI 02903 USA; 3 Department of Hygiene and Epidemiology, University of Ioannina School of Medicine, Ioannina, Greece

## Abstract

**Background:**

Conducting systematic reviews (“reviews”) requires a great deal of effort and resources. Making data extracted during reviews available publicly could offer many benefits, including reducing unnecessary duplication of effort, standardizing data, supporting analyses to address secondary research questions, and facilitating methodologic research. Funded by the US Agency for Healthcare Research and Quality (AHRQ), the Systematic Review Data Repository (SRDR) is a free, web-based, open-source, data management and archival platform for reviews. Our specific objectives in this paper are to describe (1) the current extent of usage of SRDR and (2) the characteristics of all projects with publicly available data on the SRDR website.

**Methods:**

We examined all projects with data made publicly available through SRDR as of November 12, 2019. We extracted information about the characteristics of these projects. Two investigators extracted and verified the data.

**Results:**

SRDR has had 2552 individual user accounts belonging to users from 80 countries. Since SRDR’s launch in 2012, data have been made available publicly for 152 of the 735 projects in SRDR (21%), at a rate of 24.5 projects per year, on average. Most projects are in clinical fields (144/152 projects; 95%); most have evaluated interventions (therapeutic or preventive) (109/152; 72%). The most frequent health areas addressed are mental and behavioral disorders (31/152; 20%) and diseases of the eye and ocular adnexa (23/152; 15%). Two-thirds of the projects (104/152; 67%) were funded by AHRQ, and one-sixth (23/152; 15%) are Cochrane reviews. The 152 projects each address a median of 3 research questions (IQR 1–5) and include a median of 70 studies (IQR 20–130).

**Conclusions:**

Until we arrive at a future in which the systematic review and broader research communities are comfortable with the accuracy of automated data extraction, re-use of data extracted by humans has the potential to help reduce redundancy and costs. The 152 projects with publicly available data through SRDR, and the more than 15,000 studies therein, are freely available to researchers and the general public who might be working on similar reviews or updates of reviews or who want access to the data for decision-making, meta-research, or other purposes.

## Background

Conducting systematic reviews requires a great deal of effort and resources to gather, organize, analyze, and interpret large amounts of information about included studies [1]. Increasingly, systematic reviewers look beyond traditional reports of studies (i.e., journal articles) and incorporate information from multiple sources, such as study registries, clinical study reports, conference abstracts, and communications with study authors [2, 3]. Making all data extracted during systematic reviews publicly available could offer many benefits, including reducing unnecessary duplication of effort, standardizing data, supporting analyses to address secondary research questions, and facilitating methodologic research related to both primary studies and systematic reviews (“meta-research,” i.e., methodologic and other types of research on research [4]) [5]. Examples of meta-research include research that has examined the empirical evidence for the impact of methodologic aspects of studies, such as allocation concealment [6] and outcome reporting [7].

To realize the potential benefits of public access to extracted study data from systematic reviews, we need infrastructure that supports such access. One platform for making systematic review data publicly available is the Systematic Review Data Repository (SRDR). Launched in 2012, SRDR (recently updated to SRDR+, available at https://srdrplus.ahrq.gov) is a free, Web-based, open-source, data management and archival platform for systematic reviews [8, 9]. SRDR is a relational database that allows the creation of flexible data extraction forms for structured data collection and risk of bias assessment. We, at the Brown University Evidence-based Practice Center (EPC), developed and are continuing the advancement and management of SRDR. SRDR, started by the Tufts Medical Center EPC, has been continually funded by the US Agency for Healthcare Research and Quality (AHRQ).

SRDR includes projects related to systematic reviews of any topic in any field, regardless of whether the review’s focus is on interventions, diagnosis, epidemiology, methodology, other topics, or indeed non-health-related research. The research team working on a given project in SRDR can request that the data be made available publicly. Publication is typically requested after completion of a project, but can be done even prior to completion, in which case the data that the research team changes subsequent to publication are updated on the SRDR published projects website automatically and instantly. The SRDR Management Team at the Brown EPC approves all requests for making SRDR project data public and manages the website that hosts the publicly available data (https://srdr.ahrq.gov/projects/published).

While SRDR is in part designed for data extraction, organization, and tabulation during systematic reviews, in this paper, we focus on its archival functionality. SRDR facilitates global collaboration and serves as a repository for archiving and sharing structured systematic review data for easy reuse, such as during systematic review updates or conduct of systematic reviews on closely related topics. Now in operation for 7 years, SRDR has accumulated a rich corpus of systematic review projects that have addressed a range of topics; a sizeable proportion of these systematic reviews have been funded by AHRQ or have been conducted by other groups that use SRDR frequently. AHRQ requires EPCs to make the data extracted during systematic reviews publicly available through SRDR.

To our knowledge, the projects in SRDR that have made their data public have not been systematically characterized. Such a characterization could help spread awareness about the contents of this resource and, thereby, help realize the potential benefits of public access to study data extracted during systematic reviews. Our specific objectives in this paper are to describe (1) the current extent of usage of SRDR and (2) the characteristics of all projects with publicly-available data on the SRDR website.

## Methods

### SRDR user and project statistics

On November 12, 2019, we ran custom MySQL queries on the SRDR database to gather descriptive statistics regarding SRDR users, projects, and website visits. A “user” is defined as a unique SRDR username and email address. A “project” is defined as a collaborative or individual enterprise among users within SRDR; a project may or may not be a systematic review. A user may have either a “public commentator account” or a “project contributor account.” The former type of account allows users only the ability to make comments on existing projects in SRDR. The latter type of account allows users to also create new projects and contribute modifications to existing SRDR projects in which they are participating. A “session” is defined as a unique visit from a user to the SRDR website; during a session, a user may open multiple projects and/or multiple pages within a project in SRDR. A “page” in SRDR is a webpage within the SRDR system.

### Information extracted about projects with publicly available data

For this analysis, we examined all projects with data made available publicly through SRDR as of November 12, 2019. We extracted the following information pertaining to each project:

- Year project was initiated in SRDR,
- Year project data were made available on the SRDR website,
- Discipline (clinical; public health; education; ecology; other),
- Primary focus of the project (interventions; diagnosis; epidemiology [exposure-outcome relationships]; epidemiology [incidence or prevalence]; methodology)—more than one option may have applied to a project,
- Primary health area addressed (mapped to International Classification of Diseases-10 [10]),
- Type of project (full systematic review; technical brief; evidence map; methods research; other)—systematic reviews and technical briefs were distinguished if the project’s record distinguished them,
- Whether the project was an AHRQ-funded EPC project, a Cochrane review, or neither,
- Whether the systematic review was registered in PROSPERO (an online registry of systematic review protocols; available at https://www.crd.york.ac.uk/prospero/),
- Whether the systematic review was an update of a previous systematic review (determined if the title or description of the project explicitly stated the review was an update, or if the titles of two reviews were the same/similar and we were otherwise aware of one being an update),
- Funding source for the project,
- Country of the corresponding author,
- Number of research questions (referred to as “Key Questions” in AHRQ-funded reviews),
- Number of included studies, and
- Format(s) in which included study data were added to SRDR. There are three possible formats: (1) *manual completion* of SRDR data extraction forms; (2) *import* of data into SRDR data extraction forms; and (3) *upload* of data as flat files (e.g., Microsoft® Excel, Adobe® PDF) directly to the project (i.e., without use of SRDR data extraction forms). A given project may have added data in more than one of these possible formats. Either manual completion or importing of data into SRDR data extraction forms results in data that are structured; uploading of data results in flat files.

Regarding registration in PROSPERO, if registration status was not provided on the “Published Projects” page of the SRDR website, we examined any available full report of the systematic review. We obtained full reports of AHRQ-funded systematic reviews from the AHRQ Website (https://www.ahrq.gov/research/findings/evidence-based-reports/index.html), Cochrane systematic reviews from the Cochrane Library (https://www.cochranelibrary.com), and other systematic reviews from journal articles. If PROSPERO registration status was not provided in the full report, we searched the PROSPERO registry (https://www.crd.york.ac.uk/prospero/#searchadvanced) using keywords from the title of the project. If these efforts did not yield a PROSPERO record for the systematic review, we considered it to be not registered in PROSPERO. Of note, PROSPERO does not allow registration of scoping reviews. Therefore, we did not examine PROSPERO registration status for technical briefs, evidence maps, and methods research.

### Data extraction process

We used SRDR to develop a standardized data extraction form that included 22 items (form available at https://bit.ly/2VhR7DU; and data from this project are available publicly at https://srdr.ahrq.gov/projects/published). Two investigators extracted and verified data (BTS and IJS). For each project, one investigator extracted all information and a second verified the data. All discrepancies were resolved through discussion.

Of note, when more than one project pertained to the same systematic review, we counted them as separate projects. Also, for each project, we extracted all information from SRDR (except for PROSPERO registration); we did not examine any available systematic review protocols, associated journal articles, or other external sources of information. As such, the information presented in this paper reflects the information in SRDR and not necessarily what might be reported elsewhere for a given systematic review.

### Statistical analyses

We analyzed frequencies and percentages for categorical items and medians and interquartile ranges (IQRs) for continuous items. We compared categorical items descriptively. We performed Kruskal-Wallis tests for hypothesis testing of differences between medians of continuous items. We performed all analyses using Stata® version 16 (College Station, Texas, USA).

## Results

### SRDR user and project statistics

As of November 12, 2019, SRDR has had 2552 individual user accounts belonging to users from 80 countries. These include 1407 project contributor accounts from 53 countries; the remaining accounts are public commentator accounts. Countries with the most project contributor accounts are the USA, Canada, the UK, and Greece. From January 1 to November 12, 2019, the SRDR website had an average of 84 sessions per day and 70 visitors per day; each visitor visited approximately 4.6 pages per session. All told users have created 735 projects in SRDR, with a median of three collaborators per project (range 1 to 54). The total number of studies across all these 735 projects is 90,142.

### Statistics related to projects with publicly available data

**Fig. 1:**
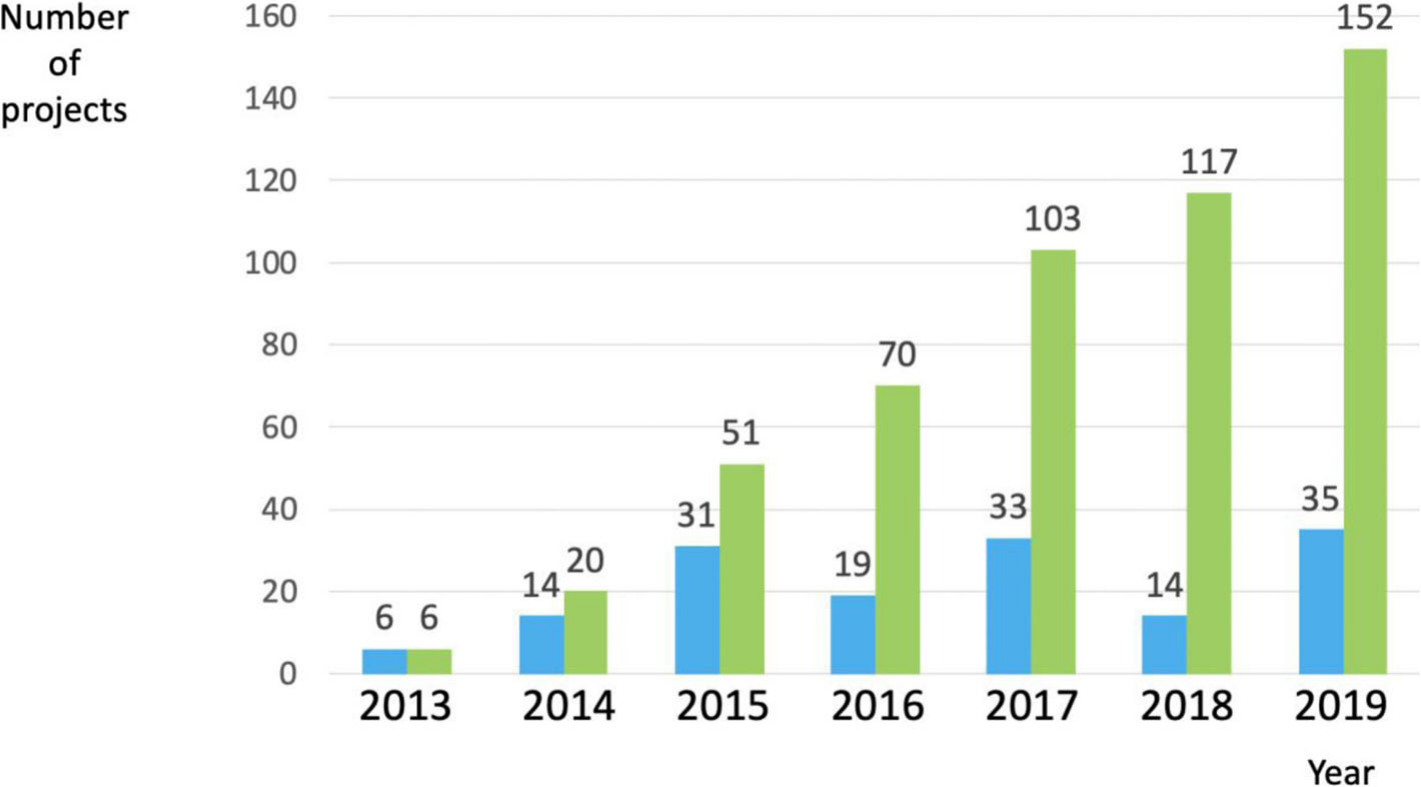
Annual and cumulative numbers of projects with publicly available data on the Systematic Review Data Repository (SRDR) website since the year after SRDR’s inception in 2012 (i.e., 2013 to 2019). Note: Data for 2019 only includes January 1, 2019 to November 12, 2019. The spike in the number of projects in 2019, although it includes data from only approximately 10.5 months is because we recently reached out to leads of all existing projects in SRDR to encourage them to make the project data available publicly. Blue bars = annual number of projects. Green bars = cumulative number of projects

We found that data are available publicly for 152 of the 735 projects in SRDR (21%). Considering full years since the 2012 launch of SRDR (i.e., 2013 to 2018), data from 25.3 projects per year, on average, have been made available publicly through SRDR (Fig. 1). The median time from project initiation in SRDR to public availability of the data through SRDR was 4 months (IQR 1 to 14). Note that this does not include the time spent on the systematic review steps before data extraction (e.g., question formulation, abstract screening) that have traditionally occurred outside SRDR.

The 152 published projects pertain to 148 distinct systematic reviews. One review was associated with four projects and another review was associated with two projects; in both instances, the multiple projects pertained to separate research questions of the same review. Three reviews each included one or more updated reviews, corresponding to seven SRDR projects. Because each update involved non-trivial changes to the research questions, we considered these seven projects as seven separate systematic reviews. In this paper, we consider projects as the unit of analysis. All told, the 152 projects include data from 15,621 studies (not accounting for overlap of some studies between projects).

### Descriptive characteristics of projects with publicly available data

Most of the published projects are in clinical fields (144/152 projects; 95%); the remainder are in public health (6/152 projects; 4%) or toxicology (2/152 projects; 1%) (Table 1). The primary focus of most projects is either interventions (therapeutic or preventive) (109/152 projects; 72%) or diagnosis (15/152 projects; 10%). The projects address a range of health areas. The most frequent health area addressed is mental and behavioral disorders (31/152 projects; 20%); the next most frequent is diseases of the eye and ocular adnexa (23/152 projects; 15%). For comparison, the most frequent health areas addressed in the 588 projects without publicly available data are diseases of the eye and ocular adnexa (85/588 projects; 15%); endocrine, nutritional, and metabolic diseases (50/588 projects; 9%); and neoplasms (37/588 projects; 6%). Appendix lists the specific topics addressed in all 152 projects with publicly available data, by health area.

**Table 1:** Projects with data available publicly on the Systematic Review Data Repository (SRDR) website as of November 12, 2019, sorted by whether or not the review was funded by the Agency for Healthcare Research and Quality (AHRQ)

Characteristic	Projects funded by AHRQ (*N* = 104)		Projects not funded by AHRQ (*N* = 48)		All projects (*N* = 152)	
	*N*	%	*N*	%	*N*	%
Discipline						
Clinical	97	(93)	47	(98)	144	(95)
Public health	5	(5)	1	(2)	6	(4)
Toxicology	2	(2)	0	(0)	2	(1)
Primary focus of the review						
Interventions (therapeutic or preventive)	79	(76)	30	(63)	109	(72)
Diagnosis	14	(14)	1	(2)	15	(10)
Epidemiology—exposure-outcome association	0	(0)	10	(21)	10	(7)
Epidemiology—incidence or prevalence	2	(2)	2	(4)	4	(3)
Prognosis	1	(1)	0	(0)	1	(1)
Screening	6	(6)	0	(0)	6	(4)
Methodology	2	(2)	5	(10)	7	(5)
Primary health area addressed (per International Classification of Diseases-10)						
Mental and behavioral disorders	30	(29)	1	(2)	31	(20)
Diseases of the eye and ocular adnexa	1	(1)	22	(46)	23	(15)
Neoplasms	11	(11)	2	(4)	13	(9)
Diseases of the musculoskeletal system and connective tissue	11	(11)	1	(2)	12	(8)
Symptoms, signs, and abnormal clinical and laboratory findings, not elsewhere classified	4	(4)	6	(13)	10	(7)
Diseases of the genitourinary system	5	(5)	4	(8)	9	(6)
Diseases of the circulatory system	7	(7)	1	(2)	8	(5)
Diseases of the respiratory system	7	(7)	0	(0)	7	(5)
Certain infectious and parasitic diseases	7	(7)	0	(0)	7	(5)
Factors influencing health status and contact with health services	5	(5)	1	(2)	6	(4)
Injury, poisoning, and certain other consequences of external causes	5	(5)	0	(0)	5	(3)
Endocrine, nutritional, and metabolic diseases	2	(2)	2	(4)	4	(3)
Pregnancy, childbirth, and the puerperium	3	(3)	1	(2)	4	(3)
Diseases of the digestive system	2	(2)	1	(2)	3	(2)
Congenital malformations, deformations, and chromosomal abnormalities	1	(1)	0	(0)	1	(1)
Diseases of the ear and mastoid process	1	(1)	0	(0)	1	(1)
Codes for special purposes	0	(0)	1	(2)	1	(1)
Other—methodology	2	(2)	5	(10)	7	(5)
Type of project						
Full systematic review/technology assessment	93	(90)	39	(81)	132	(87)
Technical brief	8	(8)	0	(0)	8	(5)
Methods research	2	(2)	5	(10)	7	(5)
Evidence map	1	(1)	4	(8)	5	(3)
Registration status in PROSPERO (for full systematic reviews/technology assessments only)						
Yes	46	(49)	12	(31)	48	(44)
No	47	(51)	27	(69)	74	(56)
Funding source(s) for project (more than one funding source could apply)						
Government	104	(100)	32	(70)	136	(91)
Pharmaceutical industry	0	(0)	0	(0)	0	(0)
Other industry	0	(0)	5	(11)	5	(3)
Foundation/professional society	0	(0)	3	(7)	3	(2)
Other funding source	0	(0)	11	(24)	11	(7)
Explicitly stated that no funding was received	0	(0)	2	(5)	2	(2)
Country of corresponding author						
USA	102	(98)	43	(90)	145	(95)
Canada	2	(2)	0	(0)	2	(1)
UK	0	(0)	2	(4)	2	(1)
Germany	0	(0)	1	(2)	1	(1)
Lebanon	0	(0)	1	(2)	1	(1)
Saudi Arabia	0	(0)	1	(2)	1	(1)
Format of addition of included study data to SRDR (more than one format could apply)						
Manual completion of SRDR data extraction forms	27	(26)	39	(81)	66	(43)
Import of data into SRDR data extraction forms	23	(22)	19	(40)	42	(28)
Upload of data as flat files (i.e., not into SRDR data extraction forms)	68	(65)	2	(4)	70	(46)

Most projects are full systematic reviews (132/152 projects; 87%). Fewer than half of these (48/117 projects; 44%) were registered in PROSPERO. Among all 152 projects, most (91%) were funded by government sources and 95% have a US-based corresponding author. The data in almost two-thirds of all projects (94/152 projects; 62%) are structured, while the remainder are in flat files (58/152 projects; 38%).

### Comparison between characteristics of AHRQ-funded and non-AHRQ-funded projects with publicly available data

About two-thirds of the projects (104/152; 68%) were funded by AHRQ. There are some notable differences between projects funded by AHRQ and those that were not (Table 1). Approximately one in five non-AHRQ-funded projects (21%) focus on exposure-outcome relationships, while none of the AHRQ-funded projects do. Almost 3 in 10 AHRQ-funded projects (29%) focus on mental and behavioral disorders, while only 2% of the non-AHRQ-funded projects do. Instead, almost half of the non-AHRQ-funded projects (46%) focus on diseases of the eye and ocular adnexa, largely due to the fact that Cochrane Eyes and Vision, based in London, UK, with a Satellite in Baltimore, Maryland, USA, has been encouraging its systematic review authors to use SRDR for data extraction. While all AHRQ-funded projects were government-funded, 3 in 10 non-AHRQ-funded projects (30%) were funded by non-government sources. Almost half of the non-AHRQ-funded projects (23/48; 48%) are Cochrane systematic reviews.

Overall, the 152 projects address a median of three research questions each (IQR 1–5) (Table 2). AHRQ-funded projects address a considerably higher number of research questions than non-AHRQ-funded projects (median 4 vs. 1; *P* = 0.0001). Overall, the 152 projects include a median of 70 studies each (IQR 20–130). AHRQ-funded projects include more than six times as many studies as non-AHRQ-funded projects (median 84 vs. 13.5; *P* = 0.0001).

**Table 2:** Numbers of research questions and included studies in projects with data available publicly on the Systematic Review Data Repository (SRDR) website as of November 12, 2019, sorted by whether or not the review was funded by the Agency for Healthcare Research and Quality (AHRQ)

Characteristic	Projects funded by AHRQ (*N* = 104)	Projects not funded by AHRQ (*N* = 48)	All projects (*N* = 152)
Number of research questions per project			
Median (interquartile range)	4 (3 to 6)	1 (1 to 1)	3 (1 to 5)
Range	1 to 15	1 to 5	1 to 15
Number of included studies per project^a^			
Median (interquartile range)	84 (36 to 139)	13.5 (3 to 58)	70 (20 to 130)
Range	0 to 281	1 to 2013	(0 to 2013)

^a^Excludes three projects that examined systematic reviews only (i.e., did not examine primary studies)

## Discussion

Since its inception in 2012, SRDR has accumulated a corpus of 152 systematic review projects with publicly available data from more than 15,000 studies. Data from these projects and studies can be accessed by anyone around the world to review, re-use in a new systematic review or related research project, conduct methodologic research, or otherwise use for various purposes. Almost two-thirds of these projects include data in a structured format. A majority of the 152 projects are in clinical fields, focus on interventions or diagnosis, and are funded by government sources. The projects cover various health areas, with mental and behavioral disorders and diseases of the eye and ocular adnexa being the most common.

### Comparison with other investigations

There are some interesting differences between the findings of this investigation and others that have examined the characteristics of systematic reviews in health. For example, others have found that about half of systematic reviews have focused on interventions and that about 45% have been funded by government sources [11, 12]. In SRDR, 72% of publicly available reviews focused on interventions and 91% were funded by government sources. These higher proportions in SRDR are largely driven by the large proportion of reviews in it that have been funded by AHRQ (68%) or are Cochrane systematic reviews (15%). AHRQ-funded and Cochrane systematic reviews tend to focus more on interventions than do other systematic reviews. Similarly, another investigation found that the median number of included studies per systematic review was 15 [11], whereas the median number of studies per review in SRDR is 70. This difference is also largely driven by the predominance of AHRQ-funded reviews, which tend to be broader in scope and address more research questions than non-AHRQ-funded systematic reviews. Indeed, in SRDR, the median number of research questions per AHRQ-funded review is approximately eight times that of non-AHRQ-funded reviews.

Our finding that 44% of eligible systematic reviews were registered in PROSPERO is a considerable improvement from the 4% that was reported in a random sample of 300 systematic reviews published in 2014 [11]. However, since AHRQ and Cochrane strongly encourage PROSPERO registration, we would expect reviews in SRDR to be more likely to have registered protocols than other systematic reviews. While the higher percentage is a good sign, we urge *all* systematic review teams to register their systematic reviews prospectively in PROSPERO. Prospective registration offers many benefits, such as promoting transparency, reducing the potential for bias, and reducing the potential for redundancy [13, 14]. Additionally, in light of the growing numbers of abridged types of evidence syntheses, such as evidence maps/scoping reviews [15], living systematic reviews [16], and rapid reviews/technical briefs [17], we agree with Page et al. [12] that the PROSPERO registry should expand its eligibility criteria to include these other types of reviews.

### Potential value of publicly available data from systematic reviews to the global community

To our knowledge, SRDR is one of a kind. It serves as a free, online, data management platform for collaboration among members of a systematic review team [8]. SRDR also offers free, open access to data about primary studies that have been extracted for systematic and other reviews on a range of topics. In this way, SRDR helps advance the open-access movement in science. We agree, however, with current guidance that those re-using shared data should cite the original data source (i.e., the systematic review and the SRDR platform from which the data were obtained) [5, 18]. To facilitate such citing, we provide each publicly available project in SRDR with an associated digital object identifier (DOI) for easy and persistent online identification.

The past decade has witnessed an almost threefold surge in the number of systematic reviews [11]. While some of these systematic reviews have been demonstrated to be redundant [19], conducting new systematic reviews on topics related to existing systematic reviews is often required. For example, an update to an existing systematic review may be needed if it is out of date and/or new studies are available. Other common scenarios that lead to a new systematic review being needed are when the eligibility criteria of an existing related systematic review were too narrow, a new type of intervention or comparator has emerged, or a broader search is needed [20]. In each of these and other related scenarios, a considerable amount of time and resources can be saved by using previously extracted data, where relevant. SRDR can help fulfill this need [8].

Efforts have been underway to begin strides towards a future in which data extraction for systematic reviews might be accurately and efficiently conducted using automation technologies [21–23]. In that context, while archival of data would still serve the purpose of transparency, re-use of data might not be of much added value because technology would be able to conduct data extraction inexpensively. While such a “revolution in automation of systematic reviews” is on the horizon, we are not there yet [24]. Until we arrive at such a future, one in which the systematic review and broader research communities are comfortable with the accuracy of automated data extraction, re-use of data extracted by humans can help reduce redundancy and costs.

There appears to be support for sharing systematic review data. A 2014 survey found that 83% of systematic reviewers affiliated with the Cochrane Individual Participant Data (IPD) Review Group supported it [25]. However, we acknowledge that there might be barriers to completely relying on previously extracted data, especially when the previous systematic review team is different from the team undertaking the new systematic review. There may be concerns about whether the set of items extracted from studies in the previous review is adequate and whether the data extracted are accurate. While SRDR does not guarantee the adequacy and accuracy of extracted data, these aspects can be assessed by systematic review teams, such as through examination of data from a random sample of the studies. Alternatively, systematic review teams may choose to use the previously extracted data as the initial extraction in an approach similar to single (de novo) data extraction and verification.

SRDR can serve as a valuable platform for conducting methodologic research. Examples of such research that has already been conducted using SRDR are the Data Abstraction Assistant (DAA) Trial (a randomized controlled trial that compared different data extraction approaches [26–28]) and the current study and the six other methodologic projects described in this paper [29–34]. SRDR also can serve as a source of data for meta-research (i.e., methodologic and other types of research on research [4]). For example, researchers might analyze the populations, interventions, comparators, outcomes, funding sources, and/or other factors across systematic reviews, either within or across health areas.

Accessing and downloading publicly available data from SRDR is relatively straightforward through the SRDR Published Projects Website (available at https://srdr.ahrq.gov/projects/published). As the SRDR management team, we are happy to help and/or partner with researchers to do so (see author contact information).

### Limitations to publicly available data on SRDR

While the SRDR management team encourages and helps systematic review teams to make their data public and manages the website, we do not monitor the accuracy or completeness of the data. Inaccuracies in publicly available data in SRDR occur due to errors in data extraction from reports of primary studies [35]. It is not uncommon for such errors to be corrected in the final versions of reported data (e.g., journal publications), but not in SRDR. Because SRDR is not yet a data analysis platform, systematic review teams might not be vigilant about retrospectively updating data in SRDR to fix errors that might have been detected during data cleaning or analysis after exporting the data outside SRDR to statistical software or other applications. Second, SRDR is an evolving platform that is striving to improve how best to archive systematic review data for easy re-use. For example, while SRDR offers structured data extraction forms and structured data entry, these features are not always fully taken advantage of; more than one-third of the projects in SRDR have simply uploaded data as flat files. We recently developed an improved mechanism through which systematic reviews can import data from flat files into forms in SRDR so that the data can be shared in a structured format [36]. Third, it should be acknowledged that data have been made available for only a quarter of all projects in SRDR. While we, as the SRDR management team, do not require teams of systematic reviewers to make their data available, we encourage them to do so as quickly as possible. We believe that the median time from SRDR project initiation (i.e., data extraction phase) to public availability of data of 4 months is satisfactory. While we do not track the direction, magnitude, or statistical significance of results of systematic reviews in SRDR, a recent study by other investigators has demonstrated that statistical significance of results is not associated with the duration from protocol registration in PROSPERO to journal publication (for non-Cochrane systematic reviews) [37].

It should be noted that, since 2015, AHRQ has required EPCs to make each of their AHRQ-funded review’s data available publicly through SRDR upon completion of the review. For systematic reviewers working on non-AHRQ-funded projects, there is less of an incentive to make data available publicly. We strongly agree with Wolfenden and colleagues that platforms, such as SRDR, that make systematic review data available publicly can help maximize returns on the significant investments that are made in the systematic review enterprise [5]. We urge more systematic review teams to make their data available publicly.

### Limitations to this study

The characteristics of the systematic reviews reported in this study are not intended to be representative of all systematic reviews. As discussed, most of the systematic reviews are AHRQ-funded or Cochrane reviews, leading to a predominance of reviews addressing clinical fields, evaluating interventions, and including higher numbers of studies than has been demonstrated in cross-sections of systematic reviews in other investigations.

## Conclusions

We have described the characteristics of 152 systematic review projects with data that are available publicly through SRDR. These projects, and the more than 15,000 studies therein, are freely available to researchers and the general public who might be working on similar systematic reviews or updates of systematic reviews or who want access to the data for decision-making, meta-research, or other purposes.

## Data Availability

The data gathered during this project are available publicly through https://srdr.ahrq.gov/projects/1324.

## Appendix

**Table 3:** Topics of the 152 projects with publicly available data on the Systematic Review Data Repository (SRDR) website as of November 12, 2019, categorized by health area

Health area (number of projects)	Topic
Mental and behavioral disorders (31 projects)	Adverse behavioral effects of caffeine consumption
	Age-related cognitive decline, mild cognitive impairment, and clinical Alzheimer’s-type dementia
	Aggressive behavior in psychiatric patients
	Agitation and aggression in dementia
	Antipsychotics for children and young adults
	Anxiety in children
	Attention deficit hyperactivity disorder (ADHD) in children and adolescents
	Atypical antipsychotics
	Autism spectrum disorder
	Autism spectrum disorder
	Autism spectrum disorder
	Autism spectrum disorder
	Binge-eating disorder
	Bipolar disorder
	Developmental disabilities, intellectual disability, and autism spectrum disorder
	Disparities within serious mental illness
	Disruptive behavior in children and adolescents
	Insomnia disorder
	Major depressive disorder
	Major depressive disorder
	Major depressive disorder
	Major depressive disorder
	Major depressive disorder in adults
	Major depressive disorder in older adults
	Management strategies to reduce psychiatric readmissions
	Mental health care for children and adolescents
	Opioid use disorder
	Post-acute coronary syndrome depression
	Posttraumatic stress disorder (PTSD)
	Schizophrenia
	Treatment-resistant depression
Diseases of the eye and ocular adnexa (23 projects)	Acanthamoeba keratitis
	Active trachoma
	Active trachoma
	Bacterial keratitis
	Cataract
	Cataract and glaucoma
	Central serous chorioretinopathy
	Chronic angle closure glaucoma
	Chronic non-infectious uveitis
	Glaucoma
	Glaucoma
	Glaucoma
	Glaucoma
	Intermittent exotropia
	Neovascular age-related macular degeneration
	Neovascular age-related macular degeneration
	Onchocerciasis
	Optic neuritis
	Pigmentary glaucoma
	Primary congenital glaucoma
	Retinal prostheses
	Retinitis pigmentosa
	Trachoma trichiasis
Neoplasms (13 projects)	Basal cell and squamous cell carcinoma of the skin
	Basal cell carcinoma
	Decision aids for cancer screening and treatment
	Hepatocellular carcinoma
	Infantile hemangioma
	Lymphoma, colorectal cancer, and head and neck cancer
	Metastatic breast cancer
	Non-metastatic muscle-invasive bladder cancer
	Non-muscle-invasive bladder cancer
	Pancreatic adenocarcinoma
	Primary breast cancer
	Risk assessment, genetic counseling, and genetic testing for BRCA-related cancer in women
	Small cell lung cancer
Diseases of the musculoskeletal system and connective tissue (12 projects)	Acute pain
	Adverse bone and calcium balance effects of caffeine consumption
	Chronic pain
	Common hip fractures
	Early rheumatoid arthritis
	Fibromyalgia
	Fractures in men and women with low bone density or osteoporosis
	Low-back pain
	Low-back pain
	Lower limb prosthesis
	Osteoporosis fracture prevention
	Primary and secondary osteoarthritis of the knee
Symptoms, signs, and abnormal clinical and laboratory findings, not elsewhere classified (10 projects)	Acute adverse effects of caffeine consumption
	Dietary fiber and health
	Dietary sugars and health
	Home-based primary care interventions
	Low calorie sweeteners
	Low calorie sweeteners
	Palliative care
	Routine preoperative testing
	Silicone gel breast implants
	Total Worker Health® (TWH)
Diseases of the genitourinary system (9 projects)	Genitourinary complaints in post-menopausal women
	Benign prostatic hyperplasia
	Chronic kidney disease
	Infertility
	Polycystic ovary syndrome
	Renal transplantation
	Stress urinary incontinence in women
	Urinary incontinence in adult women
	Uterine fibroids
Diseases of the circulatory system (8 projects)	Adverse cardiovascular effects of caffeine consumption
	Atrial fibrillation
	Coronary artery disease
	Lower extremity chronic venous disease (LECVD)
	Renal artery stenosis
	Stroke prevention in patients with atrial fibrillation
	Venous thromboembolism prophylaxis
	Venous thromboembolism prophylaxis in orthopedic surgery
Certain infectious and parasitic diseases (7 projects)	*Clostridium difficile* infection
	*Clostridium difficile* infection
	Healthcare-associated infections
	Pre-exposure prophylaxis for prevention of HIV infection
	Screening for hepatitis B virus infection in pregnant women
	Screening for HIV infection in asymptomatic, nonpregnant adolescents and adults
	Screening for HIV infection in pregnant women
Diseases of the respiratory system (7 projects)	Acute exacerbations of chronic obstructive pulmonary disease (COPD) in adults
	Acute respiratory tract infections
	Asthma
	Asthma
	Asthma
	Asthma
	Obstructive sleep-disordered breathing or recurrent throat infection in children
Factors influencing health status and contact with health services (6 projects)	Health disparities
	Health information exchange
	Health services
	Safety in nursing home settings
	Telehealth
	Telehealth
Injury, poisoning, and certain other consequences of external causes (5 projects)	Field triage of trauma
	Physiologic predictors of severe injury
	Suspected opioid overdose
	Screening for elevated blood lead levels in children
	Screening for elevated blood lead levels in pregnant women
Endocrine, nutritional, and metabolic diseases (4 projects)	Diabetes
	Diabetes
	Diabetes
	Type 2 diabetes
Pregnancy, childbirth, and the puerperium (4 projects)	Adverse reproductive and developmental effects of caffeine consumption
	Breastfeeding programs, policies, uptake and maternal health outcomes in developed countries
	Gestational diabetes
	Postpartum hemorrhage
Diseases of the digestive system (3 projects)	Bariatric surgery in the Medicare population
	Fecal incontinence
	Non-alcoholic fatty liver disease (NAFLD)
Congenital malformations, deformations, and chromosomal abnormalities (1 project)	Ankyloglossia
Diseases of the ear and mastoid process (1 project)	Tympanostomy tubes in children
Codes for special purposes (1 project)	Privacy and genetic information
Other—methodology (7 projects)	Decision and simulation modeling
	Examination of loss of information related to outcome choice in meta-analyses
	Indexing of SRDR projects (project for the current study)
	Reliability of systematic reviews addressing corneal diseases
	Summary of outcomes in clinical trials addressing HIV/AIDS
	Summary of outcomes in clinical trials in ophthalmology
	Summary of outcomes in systematic reviews addressing dry eye

## Abbreviations

AHRQ: Agency for Healthcare Research and Quality
DAA: Data Abstraction Assistant
DOI: Digital object identifier
EPC: Evidence-based Practice Center
IPD: Individual patient data
IQR: Interquartile range
SRDR: Systematic Review Data Repository
